# The Role of the Motor System in L1 and L2 Action Verb Processing for Chinese Learners of English: Evidence from Mu Rhythm Desynchronization

**DOI:** 10.3390/bs14040268

**Published:** 2024-03-24

**Authors:** Yuqing Zhang, Shifa Chen, Yule Peng, Xin Yang, Junjie Yang

**Affiliations:** College of Foreign Languages, Ocean University of China, Qingdao 266100, China; zyq8995@stu.ouc.edu.cn (Y.Z.); roro0616@163.com (X.Y.); gladysyjj123@163.com (J.Y.)

**Keywords:** embodied cognition, second-language processing, Mu rhythm, event-related desynchronization (ERD), motor system

## Abstract

The nature of semantic representation has long been a key question in linguistic research. The Embodied Cognition theory challenges the traditional view of language representation, stating that semantic information stems from the sensory-motor cortex, which is activated automatically during semantic processing. However, most of the evidence comes from monolingual studies; it remains unclear whether second-language (L2) comprehension involves different semantic representations or mirrors the pattern seen in first-language (L1) processing. Therefore, the present study investigated the role of the sensory-motor system in language processing via making Electroencephalography (EEG) recordings during the processing of L1 and L2 action verbs. The results showed that L1 (Chinese) action verbs generated higher mu-event-related desynchronization (ERD) than L1 abstract verbs in the early processing stage (250 ms after verb presentation), and the same phenomenon was also observed for L2 (English). The results also indicated that language modulated the processing of action verbs, with L1 action verbs eliciting stronger ERD than L2 action verbs. These results demonstrate that the sensory-motor cortex plays a crucial role in comprehending both L1 and L2 action verbs.

## 1. Introduction

The nature of semantic representation has long been a focal point in the research fields of neuroscience, linguistics, and psychology. Traditionally, language representation has been perceived as a distinct, supplementary function of the human brain, where meaning is conveyed through abstract, amodal, and arbitrary symbols structured by syntactic rules [1,2]. However, this amodal perspective has encountered significant challenges as it cannot explain many complex problems of semantic representation, such as the encoding, storage, and extraction of language meaning [3,4]. The Embodied Cognition theory offers a convincing substitute by contending that semantic information is not abstract but rather stems from the sensory-motor system, which is activated automatically during semantic processing [3,5,6]. Word meanings are thought of as sensory-motor memory traces that are created during interactions with the external world and are similar to the perceptual or action processes that gave rise to them [7]. Therefore, it is possible to think of language comprehension as the reactivation of the corresponding experiential traces.

Over the past decades, a substantial amount of evidence indicating the involvement of the sensory-motor system in language processing has accumulated [5,8,9,10,11,12,13,14]. For instance, when individuals read action words like “kick”, “pick”, and “lick”, this process triggers the activation of brain regions associated with the preparation and execution of physical movements involving the legs, hands, and mouth [10]. Such somatotopic activations of motor areas were also found by Raposo and colleagues when participants listened to verbs related to the arms and legs, such as “grab” and “trample” [14]. Subsequent functional magnetic resonance imaging (fMRI) studies have confirmed that the sensory-motor system is involved in sentence processing, suggesting that reading and understanding sentences activates brain representations linked to related visual and motor experiences [15].

Identifying the role of the sensory-motor system in semantic processing is difficult due to the limited temporal resolution of fMRI [16,17]. One viewpoint suggests that the activation of the sensory-motor system happens after one has actually comprehended the meaning of a word or phrase, thus being epiphenomenal with respect to language comprehension (i.e., playing an epiphenomenal role) [16,18]. In contrast, others contend that the sensory-motor system has a functional role in the retrieval of lexical–semantic information, with words deriving their meanings from the automatic reactivation of past real-world experiences, thus being functional for language comprehension (playing a functional role) [19,20]. Therefore, several studies have attempted to differentiate the roles of the sensory-motor system by examining the timing of sensory-motor activations during language comprehension [21,22,23,24,25,26]. For instance, Vukovic et al. [26] conducted experiments where they applied repetitive transcranial magnetic stimulation (rTMS) to the primary motor cortex and then tested responses to different types of words (hand action verbs vs. abstract verbs) in both semantic and lexical decision tasks. The findings demonstrated a left-lateralized and meaning-specific modulation of reaction times during a semantic judgement task when rTMS was applied within 200 ms of a word’s presentation. This modulation was characterized by a decreased response speed with respect to hand-related action words and an increased response speed for abstract words. The results were interpreted as evidence of the motor cortex’s functional role in language understanding. In the investigation conducted by van Elk et al. [25], an early engagement of the motor region, evidenced by the event-related desynchronization (ERD) of the mu rhythm (for details on mu rhythms, see below), was observed before semantic processing (about 400 ms post verb presentation) and persisting for approximately 700 ms after verb presentation. It was determined by the authors that motor activation was more likely associated with lexical–semantic retrieval and integration rather than post-lexical motor imagery due to its early presentation. In another study, Boulenger, Shtyrov, and Pulvermüller [21] demonstrated quick (within 200 ms) and somatotopic engagement of the sensory-motor system during language comprehension by employing distributed source reconstruction algorithms. Comparable results were also observed in other related studies [27,28,29]. The rapidity and automatic nature of these sensory-motor activations suggest that they are probably more than just effects of language comprehension; rather, they appear to be essential for semantic processing [17].

While these electrophysiological studies provide insight into the nature of semantic representations, they are primarily concentrated on L1. It remains open to debate, however, whether L2 comprehension involves different semantic representations or mirrors the sensorimotor activations seen in L1. During the acquisition of our L1, we frequently encounter a word while simultaneously experiencing its real-world referent [7]. For instance, children often encounter the term “grasp” in situations where they use their hands to hold objects, thereby creating a direct connection between language and physical actions. In contrast, L2 learning, especially for late L2 learners, usually takes place in educational settings through symbol manipulation and direct translation from a given native language [30]. Although some very young children may not require their L1 translation equivalents as intermediaries in acquiring an L2, the association between the L2 and its corresponding physical experience may gradually diminish due to limited real-life contexts for using L2 and an extensive period of exam-oriented education from primary school to high school. Therefore, it is possible that due to later acquisition and less exposure, typical L2 speakers may use different cognitive mechanisms to process action-related language [31,32,33]. Exploring this question is of utmost importance in that if understanding L2 action-related words does not rely on the engagement of the motor cortex, then it might challenge the notion that the sensory-motor system is essential for semantic comprehension [17].

Recently, relevant studies have set out to explore the embodiment of L2 and yielded a diverse picture [34,35,36,37,38,39,40,41,42]. In some behavioral studies, indicators like reaction times and error rates in word-image matching, Stroop, go/no-go, or sentence judgment tasks have been used to validate the embodiment of L2 [40,43,44,45]. Nonetheless, these findings were derived exclusively from behavioral data, leaving the underlying mechanisms and neurocognitive bases unexamined [46,47]. Subsequent investigations employing fMRI have explored the embodiment of L2 processing from a neuroanatomical view and yielded inconclusive results [35,38,39,42]. De Grauwe et al. [35] found that action verbs (e.g., take, throw, etc.) trigger greater activation in the motor and somatosensory areas (e.g., the left postcentral gyrus) for both L1 and L2. Conversely, another study conducted by Tian et al. [39] examined hand- or arm-related literal and metaphorical phrases in L1 and L2 and found overall greater activation of sensory-motor areas during L2 processing as opposed to that for L1 [39].

In addition to these fMRI studies, which only explored the static activation of the sensory-motor system, it is of vital importance to further explore the embodiment of L2 processing from the perspective of processing a time course. One effective approach is to examine the mu rhythms revealed via EEG during action-related language processing, which is an active field within the framework of embodied cognition research [48]. Previous research [49,50,51] has defined mu rhythms as 8–12 Hz oscillations originating in the motor and premotor cortex. These rhythms offer a detailed view into the timing of motor activation. The ERD or suppression of mu rhythms can be observed when participants perform actions [52], observe other’s actions [49,53], or imagine actions [54], particularly manual actions [55,56]. Additionally, van Elk et al. [25] reported that mu oscillations can also be used to measure motor activation triggered by linguistic stimuli.

Given this evidence, the ERD technique, with its millisecond precision, emerges as an optimal method for exploring the function of the sensory-motor system in action-related language comprehension. To date, several studies have employed this technique [17,25,48,57,58], but the majority of them involved L1 speakers. Only one study, conducted by Vukovic and Shtyrov [17], has explored the embodiment of L2 action-related language processing at the word-level, finding an early (beginning around 150 ms) mu ERD when processing action-related verbs in both L1 and L2, which was interpreted as reflecting the functional role of the sensory-motor system even in non-simultaneous bilinguals. Thus, the role of the sensory-motor system in L2 semantic representations is largely unexplored in the ERD domain.

Therefore, in the present study, EEG data were obtained from the participants via recording as they read action-related verbs in both their L1 (Chinese) and L2 (English), aiming to clarify the functional role of the sensory-motor system in language processing. Based on prior theories and research on embodied cognition, our initial hypothesis posited that the activation of the motor cortex would be observed during comprehension of both L1 and L2 action verbs. If this activation occurred in the early stages (before 400 ms after verb presentation) of action verb processing, it would indicate that the involvement of the sensory-motor system, as part of the semantic access, is essential for the semantic understanding of action verbs [23,26]. Alternatively, if it occurred in the late stage (after semantic access) of action verb processing, this might show that the activation of the sensory-motor system is just a byproduct of language comprehension. As mentioned above, L1 action verbs may exhibit a stronger association with the sensory-motor system due to the differences between L1 and L2 acquisition. In addition, previous studies have firmly proved that the neural activity caused by action execution and observation is modified differently depending on personal experience [53,59,60]. Therefore, our second hypothesis posited that the motor cortex might be more robustly activated by L1 than L2, leading to a weaker mu rhythm ERD for L2. This study aims to empirically examine the validity of these two sets of predictions.

## 2. Methods

### 2.1. Participants

Twenty-five late bilinguals (8 males; mean age = 20.8 years, SD = 1.30 years) participated in this experiment. All participants were native Chinese speakers with normal or corrected-to-normal vision and no history of neurological disorders. They were recruited from Ocean University of China, and their scores on the LexTALE [61] English vocabulary test (M = 84.92, SD = 21.24) and self-reported proficiency scores (7-point Likert scale, M = 5.01, SD = 0.71) demonstrated that they had high proficiency in English as a second language. None of the participants were simultaneous bilinguals because they all began learning English as part of their formal schooling in China (mean age of acquisition = 6.68, SD = 0.73). Three people were eliminated from further analysis due to excessive blinking and artifacts in muscle action; hence, results are presented for the remaining 22 participants. The local ethics committee approved all experimental procedures. Participants provided informed written consent before the experiment and received compensation for their time.

### 2.2. Materials

The materials used in this experiment consisted of 160 verbs, including 40 L1 action verbs (e.g., 书写, 拍手, and 画画), 40 L1 abstract verbs (e.g., 知道, 决定, and 思考), 40 L2 action verbs (e.g., writing, clapping, and drawing), and 40 L2 abstract verbs (e.g., knowing, deciding, and thinking). The L1 and L2 verbs were semantically equivalent. All the action verbs were related to hand or arm actions, as previous studies have indicated that mu rhythms are particularly sensitive to manual actions [55,56]. Alongside the 160 verbs, 80 filler nouns (40 in L2 and 40 in L2) were chosen to divert participants’ attention from the fact that the experimental materials were all verbs.

In order to minimize inter-item variability, 20 participants who did not take part in the formal EEG study took part in the pre-test. The age and L2 fluency of these participants did not statistically differ from those of the study sample. They rated the familiarity, concreteness, valence, imageability, and action-relatedness of the materials using a 7-point Likert scale (1 = very low; 7 = very high). The results are shown in Table 1.

### 2.3. Experimental Task and Procedure

In the EEG experiment, a passive word-reading scheme was employed to eliminate task demands so as to avoid deep semantic post-processing and the influence of keypresses (hand actions) on the experimental results. To ensure that participants were paying attention to the stimuli, a question was occasionally presented after 40 trials (verb + noun; 20 for L1 and 20 for L2), which corresponds to 16.7% of all items. Filler trials were excluded from the EEG data analysis.

Participants were seated in a dimly lit room designed to be both acoustically and electrically shielded. They were told to silently read the words displayed on the screen and stay as motionless as possible during the experiment. The materials were programmed using E-Prime 2.0 (Psychology Tools, Inc., Pittsburgh, PA, USA). Each trial included a 1000 ms fixation cross, 350 ms of verb presentation, and 800–1000 ms of a random-duration blank screen to serve as the intertrial. Following the words requiring a response, a simple question appeared on the screen, asking participants to decide if the word they were looking at was the same as the last word they saw. Participants were instructed to respond by pressing the “F” or “J” button on the keyboard as quickly and accurately as possible within a maximum of 3000 ms. The 240 words were divided into two lists, and the stimuli within a list were in the same language (L1 or L2). The two lists were separated by an experimental list that was completely unrelated to the present experiment and a 10 min break in order to prevent any potential interference of the code switch between L1 and L2. The stimuli of each list were presented randomly, and after every 40 trials, a pop-up screen would appear to prompt the subjects to take a short break, and the subjects would respond by pressing the button to proceed with the experiment. The order of the two lists was counterbalanced between participants. In order to familiarize the participants with the experimental procedure, 10 practice trials were run prior to the formal experiment.

### 2.4. EEG Recording and Pre-Processing

Continuous EEG was recorded using a Neuroscan Curry 8 64-channel EEG recording unit. The 64 electrodes were positioned in a Quick-Cap elastic cap according to the 10/20 system. AFz was employed as ground, and the right mastoid was used as an implicit reference during the EEG recording. The online filters used included a 50 Hz notch filter and a band-pass filter ranging from 0.1 to 30 Hz. The electrodes’ impedance was maintained below 10 kΩ. Eye blinks and movements were tracked using bipolar horizontal and vertical EOG channels through two electrode pairs: one pair placed at the external canthi and the other near the infraorbital and supraorbital areas of the right eye.

EEG data were pre-processed offline using EEGLAB [62], an open source toolbox of Matlab R2019b (Mathworks, Boston, MA, USA). First, data were re-referenced offline to the average of the left and right mastoids and filtered with a 0.1–30 Hz band-pass filter. The data were subsequently segmented into epochs ranging from −600 ms to 1000 ms relative to a word’s presentation. After visual inspection of the data, epochs with irregular noise were discarded, and bad channels were interpolated with the mean of the neighboring channels. This adjustment was necessary in only four instances, and in each case, a maximum of 3 out of 64 channels were interpolated. Finally, independent component analysis was applied to remove eye-movements (blinks and saccades) and muscular artifacts.

### 2.5. Time–Frequency (TF) Analysis

The Fast Fourier Transform was used to convert the time-domain signal into a time–frequency domain signal and calculate the integrated power in the 1–25 Hz range. Power variations were calculated with a 200 ms window size and frequency steps of 1 Hz. By averaging the power over all trials in each time–frequency bin for each individual electrode, the averaged time–frequency representations (TFRs) were calculated for each condition and subject individually. All the data underwent a baseline correction (200 ms before verb presentation) to attain the absolute difference so as to measure the power changes in the post-stimulus interval.

### 2.6. Statistical Analyses

**Figure 1:**
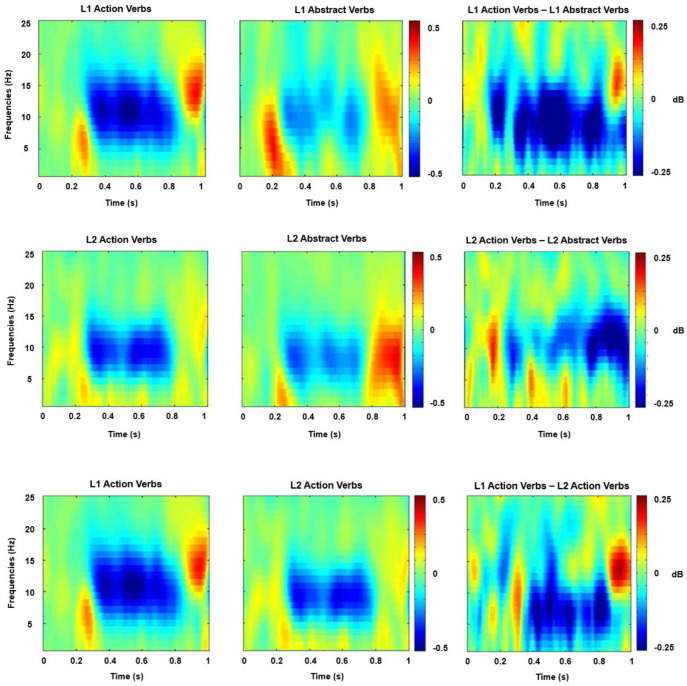
Time–frequency representation (relative to 200 ms before verb presentation) for the different experimental conditions and the three main comparisons. This figure shows the TF analysis results: The leftmost and middle columns show the mean TF representations of the L1 action verbs, L1 abstract verbs, L2 action verbs, and L2 abstract verbs for the selected electrodes throughout the sensorimotor cortex (Cz, C3, and C4) in a frequency range of 0–25 Hz. The processing of L1 and L2 action verbs elicited great suppression of mu rhythms in the time window of 250–800 ms after verb presentation. The rightmost column shows the differences between L1 action verbs and L1 abstract verbs, L2 action verbs and L2 abstract verbs, and L1 action verbs and L2 action verbs.

The suppression of mu rhythms compared to the baseline was quantified at the electrode cluster of Cz, C3, and C4. These electrode sites have been identified as the most reliable sources for detecting mu ERD originating from areas of sensorimotor cortices [58,63,64]. Our statistical analysis focused on the alpha frequency band (8–12 Hz). To objectively determine the time range for analysis, we identified intervals surrounding the ERD activity peaks from the event-related spectral perturbation (ERSP) plot (refer to Figure 1). ERSP is a technique used to assess variations in the power spectrum of the brain’s electrical activity associated with specific stimuli or events over time. In contrast to traditional EEG analysis, which mainly focuses on the amplitude of brain waves, ERSP investigates alterations in both amplitude and frequency over time, enabling a more comprehensive understanding of the brain’s dynamic responses to stimuli or events. This plot was generated by collapsing the data across all experimental conditions and averaging them among all participants. The same time window, ranging from 250 to 800 ms, was selected for this analysis. For the electrode cluster of Cz, C3, and C4 and the time–frequency window of 250–800 ms, cluster-average spectral power data were subjected to a 2 × 2 repeated measures ANOVA, with Language (L1 vs. L2) and Verb Type (action verbs vs. abstract verbs) serving as factors. Any significant interactions were further explored through separate analyses to delve into the contributions of relevant factors at every interaction level, making adjustments for multiple comparisons using Bonferroni correction (fwe = 0.05).

## 3. Results

### 3.1. Behavioral Results

The results of the behavioral task indicated that the participants were highly attentive during the experiment, with an average accuracy rate of 99% (SD = 0.39) for L1 experimental materials and 98% (SD = 0.78) for L2 experimental materials.

### 3.2. EEG Results

The results of the two-way repeated measures ANOVA revealed a significant main effect of Verb Type [F (1, 21) = 16.033, *p* = 0.001, $\eta_{p}^{2}$ = 0.433, Bonferroni-corrected]. Action verbs elicited significantly stronger ERD than abstract verbs. Moreover, a significant main effect of Language was observed [F (1, 21) = 4.428, *p* = 0.048, $\eta_{p}^{2}$ = 0.174, Bonferroni-corrected]. L1 verbs elicited significantly stronger ERD than L2 verbs irrespective of their motor-relatedness. There was a significant interaction between the factors Language (L1 vs. L2) and Verb Type (action verbs vs. abstract verbs) [F (1, 21) = 6.601, *p* = 0.018, $\eta_{p}^{2}$= 0.239, Bonferroni-corrected]. L1 action verbs generated higher ERD than L1 abstract verbs, according to a simple effect analysis of this interaction, and the same situation also occurs in L2. The results also indicated that Language modulated the processing of action verbs, with L1 action verbs eliciting stronger ERD than L2 action verbs.

## 4. Discussion

The purpose of this study was to examine the involvement of sensory-motor areas during the processing of visually presented action verbs in L1 and L2, as evidenced by mu rhythm ERD. The EEG data in response to abstract verbs and L1 and L2 action verbs were recorded in a passive reading scheme. The findings demonstrated a significant main effect of Verb Type within the 8–12 Hz mu bandwidth across the electrode cluster situated within the sensorimotor cortex, with action verbs eliciting significantly stronger ERD than abstract verbs. Additionally, a main effect of Language was observed in the sensory-motor cortex, with stronger mu rhythm ERD for L1 verbs than for L2 verbs. Most importantly, an interaction of Language and Verb Type was also found in this study, and further analysis showed that both L1 and L2 action verbs elicited stronger ERD than the corresponding L1 and L2 abstract verbs, respectively, and L1 action verbs elicited stronger ERD than L2 action verbs.

Firstly, the present study found a stronger activation of the sensorimotor system (as reflected by mu ERD) for action verbs than abstract verbs in L1 at an early stage of verb processing (250 ms post verb presentation), which is generally in line with previous research [22,23,57]. By recording high-density EEG in a passive reading task, Vukovic and Shtyrov [17] found that processing L1 action verbs elicited significant mu rhythm ERD 150 ms after the presentation of stimuli. The speed and automaticity of the activation of the sensorimotor system was interpreted by the authors as reflecting the functional role of the sensorimotor cortex in L1 action verb processing. Similarly, in a movement-priming paradigm, Mollo et al. [65] found differential brain activation in the early stages (150 ms after word presentation) of processing hand- and leg-related words, and EEG/MEG data revealed congruency effects in both the motor cortex and the posterior superior temporal cortex, the latter being a key area for language comprehension. These results suggest that pre-activating motor networks for hands and legs can differentially influence the processing of related words, highlighting the functional contributions of the sensory-motor systems to action-related language processing. In the present study, although mu rhythm ERD was identified slightly later than that of Vukovic & Shtyrov [17], it still occurred before semantic access of the action verbs [23], thus supporting the notion that the sensorimotor system plays a functional role in the processing of action language. Comparable findings were also found by Lam et al. [57], who found greater ERD of mu rhythm for action verbs compared to non-action verbs, specifically in the 300–500 ms time frame following the presentation of a verb.

Secondly, in accordance with other related studies [24,35,38,39,42], we found that the sensorimotor system was also engaged in the processing of L2 action verbs, and this engagement happened at the early processing stage. This result verifies the participation of motor systems in semantic processing and implies that the richness of L2 representations parallels that of L1, echoing the hypotheses presented by De Grauwe et al. [35]. Using fMRI, De Grauwe et al. [35] observed a notable increase in neural activation within motor and somatosensory regions for L2 motor verbs as opposed to non-motor verbs, and the authors concluded that the findings reflected the involvement of motor systems in the semantic processing of L2 action verbs. In another behavioral study, Bergen et al. [43] demonstrated that for L2 English speakers, visual images of arm and foot movements affect the processing of subsequent words (e.g., “scratch”), thereby suggesting a linkage between L2 processing and perceptual processing. These results, including ours, may indicate that despite the contrasting learning environments for L2 and L1 acquisition, with L1 typically being acquired in naturalistic settings and L2 predominantly being obtained in classroom contexts, it is evident that the processing of L2 action verbs by high-proficiency L2 speakers still necessitates the engagement of the sensorimotor system.

The embodiment of L2 can also be discussed from the perspective of the models of bilingual language presentations. While numerous models of bilingual word processing do not explicitly address embodiment effects in L2 processing, their delineations of L1 and L2 processing enable the formulation of hypotheses regarding embodiment in L2. Consistent with various well-established and contemporary models of bilingual language representations [66,67,68,69,70], there is a proposition that semantic representations of L1 and L2 can be, to a certain degree, shared and stored within a unified system. However, there are some subtle differences in the specific activation and representation patterns of L2 between various models. For example, the Bilingual Interactive Activation (BIA) and BIA+ model postulated that lexical access is indiscriminate, with lexical items being maintained in a singular, unified mental lexicon [71,72], which means that the processing of L2 can induce the automatic activation of L1 [40,73]. According to these models, if language is intrinsically embodied and based on sensorimotor processes, then these systems should be activated during both L1 and L2 usage, although the timing of this engagement might differ, potentially resulting in delayed effects in L2, as indicated by Hahne [74] and Spalek et al. [75]. However, the present study found that processing L2 action verbs elicited a significant mu rhythm ERD around 250 ms after verb presentation, consistent with the timing of L1 action verbs, which might indicate effects directly generated by L2 linguistic stimuli, independent of mediation by L1 [76]. This result would align with the Revised Hierarchical Model (RHM) developed by Kroll and Stewart [69] and Kroll et al. [77], a language production model supporting the hypothesis of selective activation. According to this model, bilinguals selectively activate either the L1 or L2 lexicon based on the context. In short, assuming mu ERD reflects motor cortex activity, this bolsters the idea that the processing of action words in both L1 and L2 depends on these regions. Consequently, it reinforces the assertions of embodied cognition theory regarding the necessity of the sensory-motor cortex in processing action-related language.

Lastly, we also observed significantly greater ERD in the processing of L1 action verbs compared to that in the processing of L2 action verbs, which was consistent with our second hypothesis of this experiment, indicating that stronger body-related experience in a native language environment correlates with enhanced involvement of the sensorimotor system in L1 language processing [17,42,53,60,78,79]. The sensorimotor integration hypothesis [80] and mechanistic models based on Hebbian principles of association learning [20] may be used to account for this asymmetrical activation of the sensorimotor system in L1 and L2 action-related verbs processing. According to Pulvermüller [20], language acquisition typically happens alongside real-world object interactions or actions. Generally, in developmental stages, a significant association is formed by correlating a word’s sound with concurrent sensorimotor experiences. While both L1 and L2 learning involve the same sensorimotor integration mechanism, the first language of a bilingual is typically used in varied, interactive real-world scenarios, fostering robust action–perception connections. In contrast, the establishment of these action–perception connections for non-simultaneous bilinguals like our subjects is more challenging due to the comparatively weaker and less rich interaction with the real world occurring when using L2. Thus, a word in L2 might share the same semantic attributes as its translation in L1, yet the activation of these attributes might be less pronounced [38]. Similar findings were obtained by Vukovic and Shtyrov [17] in another EEG study, where they discovered a stronger mu ERD for L1 action verbs compared to L2. The authors interpreted this finding as reflecting stronger modulation for the L1, which they attributed to a greater level of embodiment in L1 and a more cohesive perception–action circuit for L1 lexical–semantic representation. In addition, for the present experiment, the unique logographic structure of Chinese characters might also have caused the stronger ERD for L1 (Chinese) than for L2 (English). Most Chinese action verbs related to hand or arm movements include the 扌 radical, signifying an association with these actions (e.g., 抓/grasp and 推/push). The presence of this common radical in the materials may have triggered the larger mu ERD. Nonetheless, these results are inconsistent with those of Tian et al. [39], who found greater activation of the sensory-motor system in L2 compared with L1. The authors attributed this to the higher cognitive control demands associated with L2 processing. However, other factors must also be taken into account, including the nature of the experimental materials and the fMRI technique. Tian et al. [39] used verb–object phrases (e.g., “catch the ball”), potentially allowing for the activation of diverse semantic associations [38]. Moreover, the limited temporal resolution of fMRI may not accurately capture the timing of the sensory-motor system’s activation during language processing: while processing L2 action verbs might trigger more significant activation, such effects could be obscured.

## 5. Conclusions

In this study, we observed early motor activation during the processing of L1 and L2 action verbs by bilingual participants, as indicated by the desynchronization of the EEG mu rhythm. Additionally, L1 action verbs elicited greater mu ERD than L2 action verbs, likely due to well-developed action–perception circuits from extensive linguistic experience. We conclude that the sensory-motor cortex is essential for understanding action words and highlight the effectiveness of mu ERD in uncovering differences in neural representation between L1 and L2 action-related language.

## Figures and Tables

**Table 1:** Semantic attributes of the experimental materials.

Type	Familiarity	Concreteness	Valence	Imageability	Action Relatedness
L1 action verbs	6.23 (0.41)	5.94 (0.42)	2.91 (0.62)	6.38 (0.32)	5.99 (0.33)
L1 abstract verbs	6.27 (0.30)	2.27 (0.56)	2.70 (0.38)	2.49 (0.22)	2.31 (0.57)
L2 action verbs	6.12 (0.21)	5.76 (0.33)	2.90 (0.50)	5.97 (0.21)	5.63 (0.37)
L2 abstract verbs	6.08 (0.28)	2.23 (0.46)	3.01 (0.48)	2.56 (0.47)	2.49 (0.44)

## Data Availability

The data presented in this study are available on request from the corresponding author. The data are not publicly available due to privacy concerns.

## References

[1] Fodor J.A. (1983). The Modularity of Mind: An Essay on Faculty Psychology.

[2] Fodor J.A. (2000). The Mind Doesn’t Work That Way.

[3] Barsalou L.W. (2008). Grounded cognition. Annu. Rev. Psychol..

[4] Tyler L.K., Moss H.E. (2001). Towards a distributed account of conceptual knowledge. Trends Cogn. Sci..

[5] Fischer M.H., Zwaan R.A. (2008). Embodied language: A review of the role of the motor system in language comprehension. Q. J. Exp. Psychol..

[6] Gallese V., Lakoff G. (2005). The Brain’s concepts: The role of the Sensory-motor system in conceptual knowledge. Cogn. Neuropsychol..

[7] Zwaan R.A., Madden C.J., Pecher D., Zwaan R.A. (2005). Embodied sentence comprehension. Grounding Cognition: The Role of Perception and Action in Memory, Language, and Thinking.

[8] Fargier R., Paulignan Y., Boulenger V., Monaghan P., Reboul A., Nazir T.A. (2012). Learning to associate novel words with motor actions: Language-induced motor activity following short training. Cortex.

[9] Gianelli C., Dalla Volta R. (2015). Does listening to action-related sentences modulate the activity of the motor system? Replication of a combined TMS and behavioral study. Front. Psychol..

[10] Hauk O., Johnsrude I., Pulvermüller F. (2004). Somatotopic representation of action words in human motor and premotor cortex. Neuron.

[11] Johari K., Riccardi N., Malyutina S., Modi M., Desai R.H. (2022). HD-tDCS of primary and higher-order motor cortex affects action word processing. Front. Hum. Neurosci..

[12] Klepp A., Niccolai V., Buccino G., Schnitzler A., Biermann-Ruben K. (2015). Language-motor interference reflected in MEG beta oscillations. Neuroimage.

[13] Klepp A., Niccolai V., Sieksmeyer J., Arnzen S., Indefrey P., Schnitzler A., Biermann-Ruben K. (2017). Body-part specific interactions of action verb processing with motor behaviour. Behav. Brain Res..

[14] Raposo A., Mossa H.E., Stamatakis E.A., Tyler L.K. (2009). Modulation of motor and premotor cortices by actions, action words and action sentences. Neuropsychologia.

[15] Speer N.K., Reynolds J.R., Swallow K.M., Zacks J.M. (2009). Reading stories activates neural representations of visual and motor experiences. Psychol. Sci..

[16] Lotto A.J., Hickok G.S., Holt L.L. (2009). Reflections on mirror neurons and speech perception. Trends Cogn. Sci..

[17] Vukovic N., Shtyrov Y. (2014). Cortical motor systems are involved in second-language comprehension: Evidence from rapid mu-rhythm desynchronisation. NeuroImage.

[18] Mahon B.Z., Caramazza A. (2008). A critical look at the embodied cognition hypothesis and a new proposal for grounding conceptual content. J. Physiol. Paris..

[19] Pulvermüller F., Fadiga L. (2010). Active perception: Sensorimotor circuits as a cortical basis for language. Nat. Rev. Neurosci..

[20] Pulvermüller F. (2011). Meaning and the brain: The neurosemantics of referential, interactive, and combinatorial knowledge. J. Neuroling..

[21] Boulenger V., Shtyrov Y., Pulvermüller F. (2012). When do you grasp the idea? MEG evidence for instantaneous idiom understanding. NeuroImage.

[22] Hauk O., Pulvermüller F. (2004). Neurophysiological distinction of action words in the fronto-central cortex. Hum. Brain Mapp..

[23] Pulvermüller F., Härle M., Hummel F. (2001). Walking or talking? Behavioral and neurophysiological correlates of action verb processing. Brain Lang..

[24] Tian L., Chen H., Heikkinen P.P., Liu W., Parviainen T. (2023). Spatiotemporal Dynamics of Activation in Motor and Language Areas Suggest a Compensatory Role of the Motor Cortex in Second Language Processing. Neurobiol. Lang..

[25] van Elk M., van Schie H.T., Zwaan R.A., Bekkering H. (2010). The functional role of motor activation in language processing: Motor cortical oscillations support lexical-semantic retrieval. NeuroImage.

[26] Vukovic N., Feurra M., Shpektor A., Myachykov A., Shtyrov Y. (2017). Primary motor cortex functionally contributes to language comprehension: An online rTMS study. Neuropsychologia.

[27] Amsel B.D. (2011). Tracking real-time neural activation of conceptual knowledge using single-trial event-related potentials. Neuropsychologia.

[28] Amsel B.D., Urbach T.P., Kutas M. (2013). Alive and grasping: Stable and rapid semantic access to an object category but not object graspability. NeuroImage.

[29] Boulenger V., Roy A.C., Paulignan Y., Deprez V., Jeannerod M., Nazir T.A. (2006). Cross-talk between language processes and overt motor behavior in the first 200 msec of processing. J. Cogn. Neurosci..

[30] Li P., Jeong H. (2020). The social brain of language: Grounding second language learning in social interaction. NPJ Sci. Learn..

[31] Francis W.S., Kroll J.F., De Groot A.M.B. (2005). Bilingual semantic and conceptual representation. Handbook of Bilingualism: Psycholinguistic Approaches.

[32] Pavlenko A. (2000). New approaches to concepts in bilingual memory. Biling. Lang. Cogn..

[33] Perani D., Abutalebi J. (2005). The neural basis of first and second language processing. Curr. Opin. Neurobiol..

[34] Baumeister J.C., Foroni F., Conrad M., Rumiati R.I., Winkielman P. (2017). Embodiment and emotional memory in first vs. second language. Front. Psychol..

[35] De Grauwe S., Willems R.M., Rueschemeyer S.A., Lemhöfer K., Schriefers H. (2014). Embodied language in first- and second-language speakers: Neural correlates of processing motor verbs. Neuropsychologia.

[36] Foroni F. (2015). Do we embody second language? Evidence for ‘partial’ simulation during processing of a second language. Brain Cogn..

[37] Monaco E., Jost L.B., Lancheros M., Harquel S., Schmidlin E., Annoni J.M. (2021). First and second language at hand: A chronometric transcranial-magnetic stimulation study on semantic and motor resonance. J. Cogn. Neurosci..

[38] Monaco E., Mouthon M., Britz J., Sato S., Stefanos-Yakoub I., Annoni J.M., Jost L.B. (2023). Embodiment of action-related language in the native and a late foreign language—An fMRI-study. Brain Lang..

[39] Tian L., Chen H., Zhao W., Wu J., Zhang Q., De A., Leppänen P., Cong F., Parviainen T. (2020). The role of motor system in action-related language comprehension in L1 and L2: An fMRI study. Brain Lang..

[40] Vukovic N., Williams J.N. (2014). Automatic perceptual simulation of first language meanings during second language sentence processing in bilinguals. Acta Psychol..

[41] Xue J., Marmolejo-Ramos F., Pei X. (2015). The linguistic context effects on the processing of body–object interaction words: An ERP study on second language learners. Brain Res..

[42] Zhang X., Yang J., Wang R., Li P. (2020). A neuroimaging study of semantic representation in first and second languages. Lang. Cogn. Neurosci..

[43] Bergen B., Lau T.T.C., Narayan S., Stojanovic D., Wheeler K. (2010). Body part representations in verbal semantics. Mem. Cogn..

[44] Buccino G., Marino B.F., Bulgarelli C., Mezzadri M. (2017). Fluent speakers of a second language process graspable nouns expressed in L2 Like in their native language. Front. Psychol..

[45] Dudschig C., de la Vega I., Kaup B. (2014). Embodiment and second-language: Automatic activation of motor responses during processing spatially associated L2 words and emotion L2 words in a vertical Stroop paradigm. Brain Lang..

[46] Dudschig C., Kaup B. (2017). Is it all task-specific? The role ofbinary responses, verbal mediation, and saliency for eliciting language-space associations. J. Exp. Psychol. Learn. Mem. Cogn..

[47] Monaco E., Jost L.B., Gygax P.M., Annoni J.M. (2019). Embodied semantics in a second language: Critical review and clinical implications. Front. Hum. Neurosci..

[48] Moreno I., de Vega M., León I., Bastiaansen M., Lewis A.G., Magyari L. (2015). Brain dynamics in the comprehension of action-related language. A time-frequency analysis of mu rhythms. Neuropsychologia.

[49] Avanzini P., Fabbri-Destro M., Dalla Volta R., Daprati E., Rizzolatti G., Cantalupo G. (2012). The dynamics of sensorimotor cortical oscillations during the observation of hand movements: An EEG study. PLoS ONE.

[50] Cooper N.R., Simpson A., Till A., Simmons K., Puzzo I. (2013). Beta event-related desynchronization as an index of individual differences in processing human facial expression: Further investigations of autistic traits in typically developing adults. Front. Hum. Neurosci..

[51] Hari R. (2006). Action–perception connection and the cortical mu rhythm. Prog. Brain Res..

[52] Neuper C., Scherer R., Reiner M., Pfurtscheller G.J. (2005). Imagery of motor actions: Differential effects of kinesthetic and visual–motor mode of imagery in single-trial EEG. Cogn. Brain Res..

[53] Orgs G., Dombrowski J.H., Heil M., Jansen-Osmann P. (2008). Expertise in dance modulates alpha/beta event-related desynchronization during action observation. Eur. J. Neurosci..

[54] Llanos C., Rodriguez M., Rodriguez-Sabate C., Morales I., Sabate M. (2013). Mu-rhythm changes during the planning of motor and motor imagery actions. Neuropsychologia.

[55] Pfurtscheller G., Brunner C., Schlögl A., Lopes de Silva F.H. (2006). Mu rhythm (de) synchronization and EEG single-trial classification of different motor imagery tasks. Neuroimage.

[56] Pineda J.A. (2005). The functional significance of mu rhythms: Translating “seeing” and “hearing” into “doing”. Brain Res..

[57] Lam K.J.Y., Bastiaansen M.C.M., Dijkstra T., Rueschemeyer S.A. (2017). Making sense: Motor activation and action plausibility during sentence processing. Lang. Cogn. Neurosci..

[58] Moreno I., de Vega M., León I. (2013). Understanding action language modulates oscillatory mu and beta rhythms in the same way as observing actions. Brain Cogn..

[59] Behmer L.P., Jantzen K.J. (2011). Reading sheet music facilitates sensorimotor mudesynchronization in musicians. Clin. Neurophysiol..

[60] Quandt L.C., Marshall P.J., Bouquet C.A., Shipley T.F. (2013). Somatosensory experiences with action modulate alpha and beta power during subsequent action observation. Brain Res..

[61] Lemhöfer K., Broersma M. (2012). Introducing LexTALE: A quick and valid Lexical Test for Advanced Learners of English. Behav. Res..

[62] Delorme A., Makeig S. (2004). EEGLAB: An open source toolbox for analysis of single-trial EEG dynamics including independent component analysis. J. Neurosci..

[63] Schaller F., Weiss S., Müller H.M. (2017). EEG beta-power changes reflect motor involvement in abstract action language processing. Brain Lang..

[64] Cuellar M., Bowers A., Harkrider W.A., Wilson M., Saltuklaroglu T. (2012). Mu suppression as an index of sensorimotor contributions to speech processing: Evidence from continuous EEG signals. Int. J. Psychophysiol..

[65] Mollo G., Pulvermüller F., Hauk O. (2016). Movement priming of EEG/MEG brain responses for action-words characterizes the link between language and action. Cortex.

[66] Blanco-Elorrieta E., Caramazza A. (2021). A common selection mechanism at each linguistic level in bilingual and monolingual language production. Cognition.

[67] Dijkstra T.O.N., Wahl A., Buytenhuijs F., Van Halem N., Al-Jibouri Z., De Korte M., Rekké S. (2019). Multilink: A computational model for bilingual word recognition and word translation. Bilinguali. Lang. Cogn..

[68] Finkbeiner M., Forster K., Nicol J., Nakamura K. (2004). The role of polysemy in masked semantic and translation priming. J. Mem. Lang..

[69] Kroll J.F., Stewart E. (1994). Category interference in translation and picture naming: Evidence for asymmetric connections between bilingual memory representations. J. Mem. Lang..

[70] Van Hell J.G., De Groot A.M.B. (1998). Conceptual representation in bilingual memory: Effects of concreteness and cognate status in word association. Bilinguali. Lang. Cogn..

[71] Dijkstra T., Van Heuven W.J. (2002). The architecture of the bilingual word recognition system: From identification to decision. Bilingualism.

[72] Dijkstra T., van Heuven W.J.B., Grainger J. (1998). Simulating cross-language competition with the bilingual interactive activation model. Psychol. Belg..

[73] Costa A., Pannunzi M., Deco G., Pickering M.J. (2017). Do bilinguals automatically activate their native language when they are not using it?. Cogn. Sci..

[74] Hahne A. (2001). What’s different in second-language processing? Evidence from event-related brain potentials. J. Psychol. Res..

[75] Spalek K., Hoshino N., Wu Y.J., Damian M., Thierry G. (2014). Speaking two languages at once: Unconscious native word form access in second language production. Cognition.

[76] Kühne K., Gianelli C. (2019). Is Embodied Cognition Bilingual? Current Evidence and Perspectives of the Embodied Cognition Approach to Bilingual Language Processing. Front. Psychol..

[77] Kroll J.F., Van Hell J.G., Tokowicz N., Green D.W. (2010). The revised hierarchical model: A critical review and assessment. Bilinguali. Lang. Cogn..

[78] Calvo-Merino B., Glaser D.E., Grèzes J., Passingham R.E., Haggard P. (2005). Action observation and acquired motor skills: An FMRI study with expert dancers. Cereb. Cortex.

[79] Quandt L.C., Marshall P.J., Bouquet C.A., Young T., Shipley T.F. (2011). Experience with novel actions modulates frontal α EEG desynchronization. Neurosci. Lett..

[80] Hernandez A.E., Li P. (2007). Age of acquisition: Its neural and computational mechanisms. Psychol. Bull..

